# The Newly Isolated Endophytic Fungus *Paraconiothyrium* sp. LK1 Produces Ascotoxin

**DOI:** 10.3390/molecules17011103

**Published:** 2012-01-20

**Authors:** Abdul Latif Khan, Muhammad Hamayun, Javid Hussain, Sang-Mo Kang, In-Jung Lee

**Affiliations:** 1 School of Applied Biosciences, College of Agriculture and Life Sciences, Kyungpook National University, Daegu 702-701, Korea; Email: kmoya@hanmail.net (S.-M.K.); 2 Department of Plant Sciences, Kohat University of Science and Technology, Kohat 26000, Pakistan; Email: latifepm78@yahoo.co.uk (A.L.K.); 3 Department of Botany, Abdul Wali Khan University, Mardan 23300, Pakistan; Email: hamayun@awkum.edu.pk; 4 Department of Biological Sciences and Chemistry, College of Arts & Sciences, University of Nizwa, Nizwa 33, Oman; Email: javidhej@hotmail.com

**Keywords:** *Paraconiothyrium* sp., ascotoxin, growth inhibitory effect, endophytic fungi

## Abstract

We have isolated five endophytic fungi from the roots of *Capsicum annuum*, * Cucumis sativus* and *Glycine max*. The culture filtrates (CF) of these endophytes were screened on dwarf mutant rice (*Waito-C*) and normal rice (Dongjin-byeo). Endophyte CAC-1A significantly inhibited the growth of *Waito-C* and Dongjin-byeo. Endophyte CAC-1A was identified as *Paraconiothyrium* sp. by sequencing the ITS rDNA region and phylogenetic analysis. The ethyl acetate fraction of *Paraconiothyrium* sp. suppressed the germination of *Lactuca sativa* and *Echinochloa crus-galli* seeds. The ethyl acetate fraction of the endophyte was subjected to bioassay-guided isolation and we obtained the phytotoxic compound ascotoxin (**1**) which was characterized through NMR and GC/MS techniques. Ascotoxin revealed 100% inhibitory effects on seed germination of Echinochloa crus-galli. Compound (**1**) was isolated for the first time from *Paraconiothyrium* sp.

## 1. Introduction

Fungi are key resources for exploiting bioactive metabolites [1,2]. Among fungi, endophytes are important to screen biologically active metabolites [3]. Endophytic fungi inhabit within their host-plant without causing any disease symptoms [3]. In endophyte-host symbioses, secondary metabolites produced by endophytes contribute positively to their host [4]. Diverse classes of chemical substances like steroids, xanthones, phenols, isocoumarines, perylene derivatives, quinones, furandiones, terpenoids, depsipeptides and cytochalasines have been isolated from endophytic fungi [3,4,5]. Such substances are synthesized through polyketide pathway from mevalonate-derived C5 units and (or) using the non-ribosomal protein synthesis. A literature survey reveals that the number of novel chemical structures produced by endophytes (51%) is significantly higher than the soil fungus (38%), suggesting that these frequently overlooked endophytes are the novel source of bioactive secondary metabolites [3,4,5]. 

Furthermore, each plant species has its ecological niche [6,7], which can, either positively or negatively, regulate the growth of neighbouring plants by creating resource competition. Since, agricultural practices are the major food source for human; therefore, removing the unwanted resource competitors or weeds from crop fields is an ideal strategy for sustainable agriculture production [8,9,10,11]. The use of synthetic agrochemicals and herbicides to remove weed species is widespread and known to have devastating impacts on the agriculture and overall environment [12]. Thus, using the natural processes to improve quantity and quality of agronomics can develop the prolonged food production system [3,4,11]. Fungi in general and endophytes in particular have become an interesting natural resource for exploring bioactive substances, which can be helpful to eradicate weeds by secreting allelochemicals [6,7,8,9,10]. In the present work, we isolated endophytic fungal strains from the roots of the *Capsicum annuum, Cucumis sativus* and *Glycine max*. To assess the bioactivity, the isolated strains were screened on a dwarf phenotype of *Oryza sativa*—*Waito-C* and a normal rice Dongjin-byeo. The bioactive fungus was revealed after screening and was subjected to column chromatographic techniques, nuclear magnetic resonance (NMR) and GC/MS analysis to isolate and characterize the lettuce (*Lactuca sativa*) and weed (*Echinochloa crus-galli*) seeds inhibitory compound. 

## 2. Results and Discussion

We isolated five endophytic fungi from the roots of pepper, cucumber and soybean plants. These fungi were grown on Hagem plates for seven days. The pure cultures were grouped on the basis of colony shape, height and color of aerial hyphae, base color and surface texture [13]. The culture filtrate (CF) of these different endophytes was assayed on *Waito-C* and Dongjin-byeo rice seedlings to differentiate between growth stimulatory or inhibitory strains. The effect of CF on shoot growth and biomass of dwarf *Waito-C* and Dongjin-byeo rice seedlings were recorded after a week of treatment and the data is given in Table 1. In growth promoting strains, the CF of GMH-1B and TH-3B significantly stimulated the growth of both *Waito-C* and Dongjin-byeo as compared to sole water (DW) treated rice. In the inhibited strains, the CF of CAC-1A significantly suppressed the shoot growth and rice seedling’s biomass as compared with other strains as well as the water applied control rice seedlings (Table 1). 

**Table 1:** Effect of culture filtrate of isolated endophytes on the growth of rice seedlings.

Endophyte	*Waito-C*		Dongjin-byeo	
	Shoot length (cm)	Shoot fresh weight (g)	Shoot length (cm)	Shoot fresh weight (g)
Control (DW)	7.6 ± 0.1b	0.5 ± 0.06b	8.8 ± 0.55b	0.4 ± 0.01b
CAC-1A	6.3 ± 0.1c	0.2 ± 0.1c	7.1 ± 0.09c	0.2 ± 0.05c
CSH-5A	7.7 ± 0.2b	0.4 ± 0.05b	8.9 ± 0.34b	0.5 ± 0.05b
GMH-1B	8.6 ± 0.7a	0.8 ± 0.05a	11.8 ± 0.43a	0.7 ± 0.08b
TH-3B	8.5 ± 0.7a	0.8 ± 0.09a	11.1 ± 0.17a	0.85 ± 0.09a
CAC-1C	7.5 ± 0.2b	0.8 ± 0.09a	9.1 ± 0.77b	0.8 ± 0.07a

DW = distilled water. In each column, the different letter indicates significant (*p* < 0.05) difference as evaluated by Duncan’s Multiple Range Test (DMRT). (±) standard deviation (SD) (*n* = 5).

**Figure 1:**
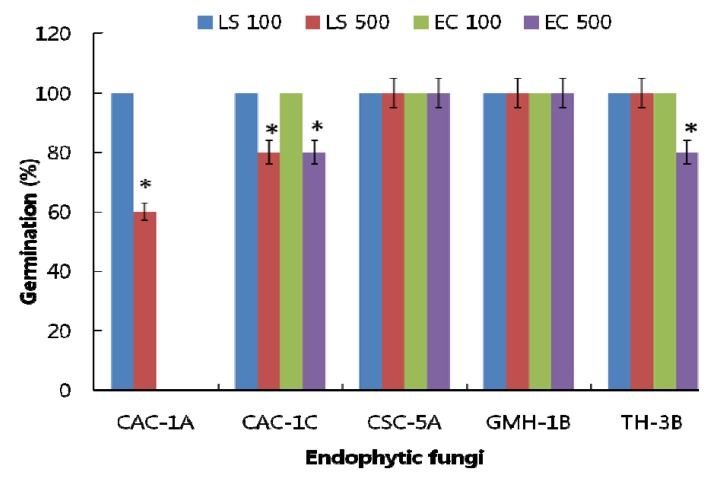
Effect of culture filtrate application of selected endophytic fungal strains on the germination of *Lactuca sativa* (LS with 100 and 500 ppm) and *Echinochloa crus-galli* (EC with 100 and 500 ppm) seeds. The ‘*’ indicates that values are significantly different (*p* < 0.05). The error bars represents ± SD (*n* = 3).

To further specify the effect of CAC-1A, we applied the CF (100 and 500 ppm dissolved in autoclaved DDW) to *Lactuca sativa* (LS) and *Echinochloa crus-galli* (EC) seeds. The rate of seed germination was recorded. The CF of CSC-5A and GMH-1B revealed 100% germination of both the seeds at 100 and 500 ppm. In case of CAC-1C and TH-3B, though all seeds (LS and EC) germinated at 100 ppm, however, at 500 ppm, the seed germination rate was 80% for both the strains (Figure 1). The CF of CAC-1A presented significant inhibitory effects to the germination of EC compared to LS. At 100 ppm, all seeds of LS germinated, however, EC seeds were suppressed at the same concentrations. Conversely, at 500 ppm, 80% of LS seeds germinated while the EC seeds were significantly suppressed and no germination was observed in comparison with the control treatments. 

**Figure 2:**
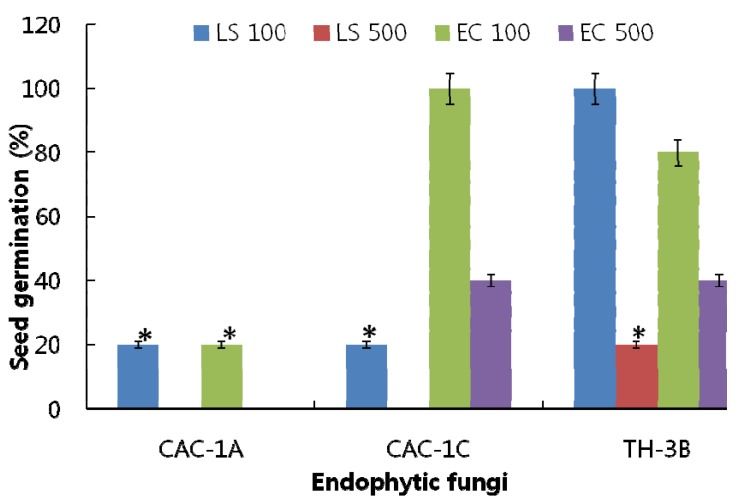
The effect of ethyl acetate extract of three fungal strains on the germination of *Lactuca sativa* (LS with 100 and 500 ppm) and *Echinochloa crus-galli* (EC with 100 and 500 ppm) seeds.The ‘*’ indicates that values are significantly different (*p* < 0.05). The error bars represents ±SD (*n* = 3).

CAC-1A, CAC-1C and TH-3B were thus selected for further ethyl acetate fractionation. After solvent-solvent partition, the resultant extracts were analyzed for their effects on seed germination of LS and EC. Two concentrations, 100 and 500 ppm were used to perform the bioassay. According to results, CAC-1A showed significantly higher inhibitory effects in comparison to TH-3B and CAC-1C and controls. The results of CAC-1A showed that at 100 ppm, the germination rate of both, LS and EC, was 20% as compared to control, while at 500 ppm, no seeds were germinated (Figure 2). The EC and LS seeds have same inhibitory response towards germination as compared to control. 

Based on the significant results of CAC-1A, the DNA was extracted from the freeze-dried mycelia and ITS (18S rDNA) region was sequenced. Phylogenetic analyses were carried out of similar sequences using MEGA 4.0 [14] and a maximum parsimony (MP) consensus tree was constructed from 13 (12 references and 1 clone) aligned partial sequences with 1 K bootstrap replications. Selected strains were run through BLASTn (BLAST nucleotide) search presenting highest sequence homology proportion and query coverage, and lowest E values. Results of BLASTn search revealed that CAC-1A has 99% sequence homology with *Paraconiothyrium* sp. In MP dendrogram, CAC-1A formed 58% bootstrap support with *Paraconiothyrium* sp. (supplementary Figure 1). On the basis of sequence homology and phylogenetic analysis results, isolate CAC-1A was identified as a new strain of *Paraconiothyrium* sp. LK1. The sequence was submitted to NCBI GenBank and was given accession No. JQ288104. 

**Figure 3:**
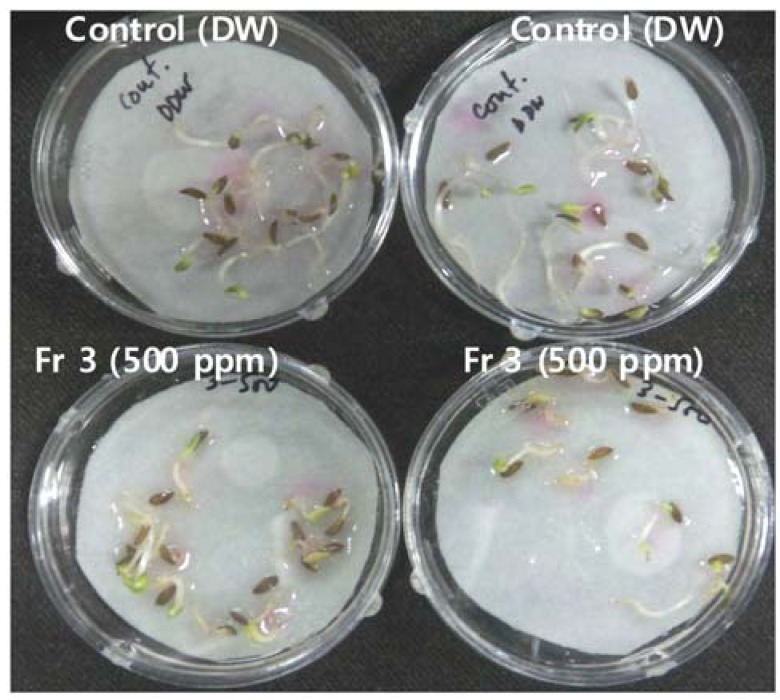
Effect of fraction 3 (500 ppm concentration) on the seed germination of LS (*n* = 5).

Since the bioactivity of *Paraconiothyrium* sp. was significant, therefore, it was subjected to column chromatography and further fractionated into six different fractions (see Experimental). Bioassay of all fractions was performed using the same 100 and 500 ppm concentrations as we used for screening. The bioassay results showed that fractions 3 and 4 were more bioactive in comparison with other fractions and control (Figure 3). However, all seeds germinated at all concentrations. 

**Figure 4:**
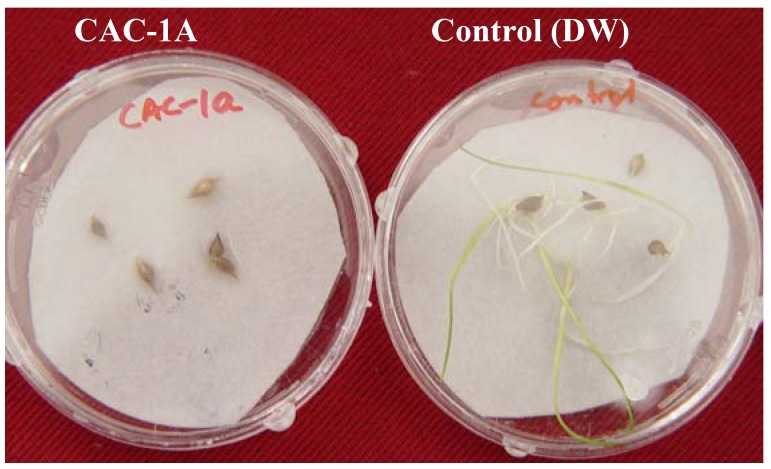
Effect of compound **1** on the growth of EC seeds (*n* = 3).

After obtaining significant bioassay results from fraction 3, it was selected for further purification through HPLC. The HPLC chromatograph showed a prominent peak of compound **1**. The ^1^H-NMR, ^13^C-NMR and GC/MS data shows that compound **1** is ascotoxin as previously reported by Vurro *et al*. [15], Ritzenthaler *et al*. [16], Wang *et al*. [17,18]. The ascotoxin was subjected to bioassay and the results showed that it is significantly inhibitory to the germination of EC seeds (Figure 4).

In the present research work, we isolated the known, phytotoxic compound ascotoxin (**1**). The bioactive compound has also been variously known as brefeldin A, decumbin, cyanein and sinergisidin [15,16,17,18]. It is a 16-membered macrolide antibiotic which had previously been isolated from a number of fungal genera: *Alternaria*, *Ascochyta*, *Penicillium*, *Curvularia*, *Cercospora*, and *Phyllosticta* [15]. It was also reported as brefeldin A from *Paecilomyces* sp. and *A. clavatus*. Ascotoxin (**1**) is a lactone antibiotic produced by fungal organisms such as *Eupenicillium brefeldianum*. Wang *et al*. [18] assessed the effects of brefeldin A (BFA) on pollen tube development in *Picea meyeri* using ﬂuorescent marker FM4-64 as a membrane-inserted endocytic/recycling marker, together with ultrastructural studies and fourier transform infrared analysis of cell walls. BFA inhibited pollen germination and pollen tube growth, causing morphological changes in a dose-dependent manner, and pollen tube tip growth recovered after transferring into BFA-free medium. Ascotoxin has several important bioactive activities, including antifungal, antiviral and anticancer properties [19]. It can even be used in weed management [15]. Furthermore, such metabolites, like those of the remaining fractions are useful for weed management, and, consequently, the search of more metabolites should be active. In this study, it is demonstrated that compound **1** may play an important role in the regulation of the weed-plant species.

## 3. Experimental

### 3.1. General

The ^1^H and ^13^C-NMR spectra were recorded in CD_3_OD using TMS as internal standard on a Bruker spectrometers operating at 400 and 500 MHz. The chemical shift values are reported in ppm (*δ* ) units and coupling constant (*J*) in Hz. For GC/MS an Agilent, 7890A-5975C with MSD equipped with JMS-HX-110 with data acquisition system and JMS-DA 500 mass spectrometer was used. High performance liquid chromatography was performed on a Shimadzu CBM-10 (Tokyo, Japan) instrument with UV/V dectector-SPD-10A, refractive index detector—RID-10A, LC-10A, which was equipped with a reversed-phase column (Luna C18 100A, 4.6 × 250 mm, Phenomenex, CA, USA). Methanol, ethyl acetate and Milli-Q water were used as elution solvents in chromatography. GC/MS was used for mass chromatography by Aligent (5975C; EI scan LOD 1 pg; carrier gas helium; ionization current 315uA, direct injection probe).

### 3.2. Isolation and Screening of Endophytic Fungus

We collected pieces of roots of *Capsicum annuum*, *Cucumis sativus* and *Glycine max* field grown plants. The roots were surface sterilized with 2.5% sodium hypochlorite (30 min in shaking incubator at 120 rpm) and washed with autoclaved distilled water (DDW) to remove any contaminants, rhizobacteria and mycorrhizal fungi. Pieces of roots (0.5 cm) were carefully placed in Petri-plates containing Hagem media (0.5% glucose, 0.05% KH_2_PO_4_, 0.05% MgSO_4_·7H_2_O, 0.05% NH_4_Cl, 0.1% FeCl_3_, 80 ppm streptomycin and 1.5% agar; pH 5.6 ± 0.2). The sterilized roots were also imprinted on separate Hagem plates to ensure the effectiveness of surface sterilization [15]. Endophytic fungi were isolated according to the method described by Khan *et al*. [19,20]. The newly emerged fungal spots were isolated and grown on potato dextrose agar (PDA) medium under sterilized conditions [14]. Total five different fungal strains were isolated and grown on PDA media. These strains were inoculated in Czapek broth (50 mL; 1% glucose, 1% peptone, 0.05% KCl, 0.05% MgSO_4_·7H_2_O, and 0.001% FeSO_4_·7H_2_O; pH 7.3 ± 0.2) and grown for seven days (shaking incubator 120 rpm; temperature 30 °C) to separate liquid culture medium and fungal mycelia (centrifugation 2,500 *g* at 4 °C for 15 min). The culture medium (culture filtrate-CF, 10 mL) and mycelium (5.4 gm) were immediately shifted to −70 °C freezer and then freeze-dried (Virtis Freeze Dryer, Gardiner, NY, USA) for 4–7 days. The lyophilized CF was diluted with 100 µL of autoclaved DDW, while the mycelia were used for genomic DNA extraction. 

Presence or absence of plant growth promoting metabolites in fungal CF was confirmed by performing screening bioassays on mutant rice cultivar *Waito-C* and normal cultivar *Oryza sativa* L. cv. Dongjin-byeo. *Waito-C* has dwarf phenotype while Dongjin-byeo has normal phenotype. For bioassay experiment, rice seeds were surface sterilized with 2.5% sodium hypochlorite for 30 min, rinsed with autoclaved DDW and then incubated for 24 h with 20-ppm uniconazol (except Dongjin-byeo) to obtained equally germinated seeds. Then pre-germinated *Waito-C* and Dongjin-byeo seeds were transferred to pots having water: Agar medium (0.8% w/v) [19] under aseptic conditions. Both the rice cultivars were grown in growth chamber (day/night cycle: 14 h, 28 ± 0.3 °C;10 h, 25 ± 0.3 °C; relative humidity 70%; 18 plants per treatment) for ten days. Ten micro-litter of fungal CF was applied at the apex of the rice seedlings [20,21]. One week after treatment, the shoot length, chlorophyll content and shoot fresh weight were recorded and compared with autoclaved DDW. Upon screening results, bioactive fungal strain CAC-1A was selected for further experiments and identification. 

### 3.3. Identification and ITS rDNA Phylogenetic Analysis

Genomic DNA was extracted from CAC-1A using the standard method of Khan *et al*. [15]. Fungal isolates were identified by sequencing the internal transcribed region (ITS) of rDNA using universal primers: ITS-1; 5′-TCC GTA GGT GAA CCT GCG G-3′ and ITS-4; 5′-TCC TCC GCT TAT TGA TAT GC-3′. The BLAST search program was used to compare the nucleotide sequence similarity of ITS region of related fungi. The closely related sequences obtained were aligned through CLUSTAL W using MEGA version 4.0 software [14] and a maximum parsimony tree was constructed using the same software. Bootstrap replications (1K) were used as a statistical support for the nodes in the phylogenetic tree.

### 3.4. Culturing Endophytic Fungi

Endophytic fungal strain, CAC-1A, CSC-5A, GMH-1B, TH-3B and CAC-1C were grown initially in Czapek broth (200 mL, 1% glucose, 1% peptone, 0.05% KCl, 0.05% MgSO_4_·7H_2_O, and 0.001% FeSO_4_·7H_2_O; pH 7.3 ± 0.2) for 7 days at 30 °C at 120 rpm. The culture filtrate of each fungus was screened on lettuce (*Lactuca sativa=* LS) and *Echinochloa crus-galli* (EC) seeds. Upon significant results, CAC-1A was selected for further analyses and studies. The Czapek culture broth containing conidia of the strain were transferred to 5 liters of Czapek broth. The broth was kept for 30 days in shaking incubator (30 °C at 200 rpm). After incubation, the supernatant and fungal mycelia were separated through centrifugation (at 5,000 *g* at 4 °C for 15 min). The supernatant was subjected to bioassay-guided isolation and mycelia were used for re-identification of the fungal strain. 

### 3.5. Extraction and Isolation

The CF was assayed for the inhibiting or promoting effect on the LS and EC seeds. Upon bioactive results of CAC-1A, TH-3B and CAC-1C, the filtrated supernatant was further extracted with an equal volume of ethyl acetate (EtOAc) three times to obtain an extract. The EtOAc extract of the CF of each strain was assayed for seed germination effects. Endophyte CAC-1A showed significant activity and the 30 days grown 5 liter culture was extracted with EtOAc three times to obtain a crude gummy extract (2.4 g). The extract was chromatographed on Luna C18 100A column (250 mm × 4.60 mm, 5 μm; Phenomenex, Torrence, CA, USA) using 60:30 MeOH in water, 100% MeOH, 60:30 MeOH in EtOAc and 100% EtOAc eluent. All six fractions were bio-assayed for their germination against both the indicator seeds. Fractions 3 (100% MeOH) and 4 (60% MeOH in EtOAc) were found to be bioactive against the germination of both the seeds. Fraction 3 (20-µL concentrated to 100 mg) was further chromatographed by HPLC using 100% MeOH (A) and water with 5% acetic acid (B) with 0–20 min (50% A); 20–40 min (80% A); 40–60 min (100% A) at a flow rate of 1.5 min/mL. The HPLC analysis provided compound **1** whose structure was elucidated and characterized using NMR and GC/MS techniques.

### 3.6. Inhibitory Bioassay

Two different concentrations of 100 and 500 ppm of each fraction of EtOAc extract were prepared by dissolving it in 1% DMSO or DW. Initial concentration was 1,000 ppm. A glass dish of 27 mm diameter with a lid and a filter paper (27 mm ø, Type Roshi Kaisha, Ltd., Tokyo, Japan) was used in the glass dish. The dilutions were applied on the filter paper and thus allowed to spread over it. Five to seven LS or EC seeds were placed on it and the dishes were sealed and packed for incubation for 72 h at room temperature. The control has only DDW or 1% DMSO solution. The experiment was repeated thrice. For each fraction, mean, SD variance [11] and standard error were calculated to determine inhibition pattern. 

### 3.7. Statistical Analysis

The significant differences among the mean values of various treatments were determined using Duncan’s multiple range tests (DMRT) at *p* < 0.05 using Statistic Analysis System (SAS 9.1, San Diego, CA, USA). To identify significant effects, the Student’s *t*-test of mean values was carried out using Graph Pad Prism software (version 5.0, San Diego, CA, USA).

## 4. Conclusions

Bioassay guided isolation from endophyte *Paraconiothyrium* sp. resulted in purification of phytotoxic compound ascotoxin (**1**). The compound suppressed the growth of *Echinochloa crus galli* seeds.

## Supplementary Materials

Supplementary materials can be accessed at: http://www.mdpi.com/1420-3049/17/1/1103/s1.

## References

[1] Dreyfuss M.M., Chapela I.H. , Gullo V.P. (1994). Potential of Fungi in the Discovery of Novel, Low-molecular Weight Pharmaceuticals. The Discovery of Natural Products with Therapeutic Potential.

[2] Proudfoot J.R. (2002). Drugs, leads and drug-likeness: An analysis of some recently launched drugs. Bioorg. Med. Chem. Lett..

[3] Schulz B., Boyle C. (2005). The endophytic continuum. Mycol. Res..

[4] Schulz B., Boyle C., Draeger S., Römmert A., Krohn K. (2002). Endophytic fungi: A source of novel biologically active secondary metabolites. Mycol. Res..

[5] Santos R.M., Rodrigues G., Fo E., Rocha W.C., Teixeira M.F.S. (2003). Endophytic fungi from *Melia azedarach*. World J. Microbiol. Biotechnol..

[6] Khan A.L., Hamayun M., Hussain J., Gilani S.A., Khan H., Kikuchi A., Watanabe K.N., Jung E., Lee I.J. (2009). Assessment of allelopathic potential of selected medicinal plants of Pakistan. Afr. J. Biotechnol..

[7] Khan A.L., Hussain J., Hamayun M., Gilani S.A., Kim Y., Rehman S., Watanabe K.N., Lee I.  (2010). Elemental allelopathy and antifungal activities of *Inula falconeri* from Himalaya Pakistan. AAS: Plant Soil Sci..

[8] Khan A.L., Hussain J., Hamayun M., Kang S.M., Watanabe K.N., Lee I.J. (2010). Allelochemical, eudesmane-type sesquiterpenoids from *Inula falconeri*. Molecules.

[9] Khan A.L., Hussain J., Hamayun M., Gilani S.A., Ahmad S., Rehman G., Kim Y.H., Kang S.M., Lee I.J. (2010). Secondary metabolites from *Inula britannica* L. and their biological activities. Molecules.

[10] Khan A.L., Hussain J., Hamayun M., Shinwari Z.K., Khan H., Kang Y.H., Kang S.M., Lee I.J. (2009). Inorganic profile and allelopathic effect of endemic *Inula koelzii* from Himalaya Pakistan. Pak. J. Bot..

[11] Hiradate S., Morita S., Sugie H., Fujii Y., Harada J. (2004). Phytotoxic *cis*-cinnamoyl glucosides from *Spiraea thunbergii*. Phytochemistry.

[12] Vyvyan J.R. (2002). Allelochemicals as lead for new herbicides and agrochemicals. Tetrahedron.

[13] Arnold A.E., Henk D.A., Eells R.L., Lutzoni F., Vilgalys R. (2007). Diversity and phylogenetic affinities of foliar fungal endophytes in loblolly pine inferred by culturing and environmental PCR. Mycologia.

[14] Tamura K., Dudley J., Nei M., Kumar S. (2007). MEGA4: Molecular evolutionary genetics analysis (MEGA) software version 4.0. Mol. Bio. Evol..

[15] Vurro M., Evidente A., Andolﬁ A., Zonno M.S., Giordano F., Motta A. (1998). Brefeldin A and a,b-dehydrocurvularin, two phytotoxins from *Alternaria zinniae*, a biocontrol agent of *Xanthium occidentale*. Plant Sci..

[16] Ritzenthaler A., Nebenführ A., Movafeghi A., Stussi-Garaud C., Behnia L., Pimpl P., Staehelin L.A., Robinson D.G. (2002). Reevaluation of the effects of brefeldin A on plant cells usingtobacco bright yellow 2 cells expressing Golgi-targeted green fluorescent protein and COPI antisera. Plant Cell.

[17] Wang J., Huang Y., Fang M., Zhang Y., Zheng Z., Zhao Y., Su W. (2002). Brefeldin A, a cytotoxin produced by *Paecilomyces* sp. and Aspergillus clavatus isolated from *Taxus mairei* and *Torreya grandis*. FEMS Immunol. Med. Microb..

[18] Wang Q., Kong L., Hao H., Wang X., Lin J., Samaj J., Baluska F. (2005). Effects of brefeldin A on pollen germination and tube growth antagonistic effects on endocytosis and secretion. Plant Physiol..

[19] Zhu J.W., Nagasawa H., Nagura F., Mohamad S.B., Uto Y., Ohkura K., Hori H. (2000). Elucidation of structural requirements of brefeldin A as an inducer of differentiation and apoptosis. Bioorg. Med. Chem..

[20] Khan A.L., Hamayun M., Kim Y.H., Kang S.M., Lee I.J. (2011). Ameliorative symbiosis of endophyte (*Penicillium funiculosum* LHL06) under salt stress elevated plant growth of *Glycine max* L. Plant Physiol.Biochem..

[21] Khan A.L., Hamayun M., Ahmad N., Waqas M., Kang S.M., Kim Y.H., Lee I.J. (2011). *Exophiala* sp. LHL08 reprograms *Cucumis sativus* to higher growth under abiotic stresses. Physiol. Plantarum.

