# Supplementary material for: The Newly Isolated Endophytic Fungus Paraconiothyrium sp. LK1 Produces Ascotoxin
Source: Molecules. 2012 Jan 20;17(1):1103–12. doi: 10.3390/molecules17011103 (PMC6268507; doi:10.3390/molecules17011103)
Supplement: Supplementary file 1 [file molecules-17-01103-s001.doc]

**Supplementary**


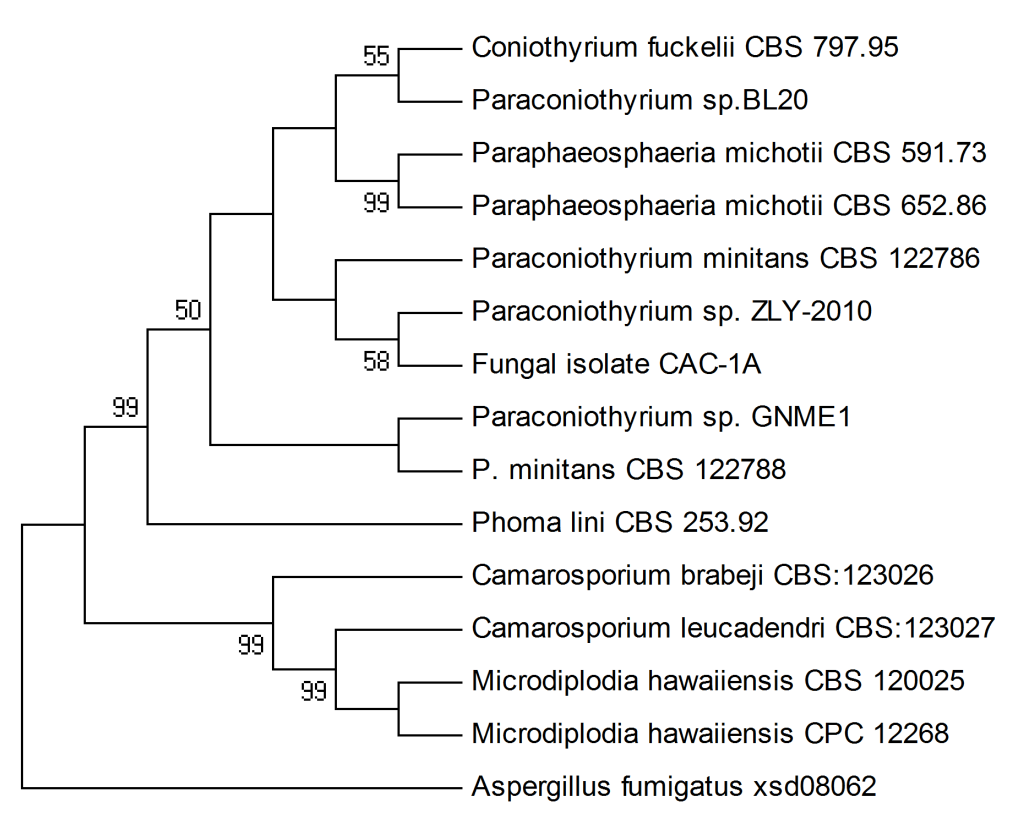


Phylogenetic tree constructed through maximum parsimony method using MEGA 4.0 [14].
The sequence obtained from ITS regions of rDNA of *Paraconiothyrium* sp. and related fungi. The bioactive endophytic fungal strain formed a sub-clade (58% bootstrap support) with *Paraconiothyrium* sp. *Aspergillus fumigatus* was taken as an out-group.
